# Perceived attractiveness of Czech faces across 10 cultures: Associations with sexual shape dimorphism, averageness, fluctuating asymmetry, and eye color

**DOI:** 10.1371/journal.pone.0225549

**Published:** 2019-11-21

**Authors:** Tomáš Kočnar, S. Adil Saribay, Karel Kleisner

**Affiliations:** 1 Department of Philosophy and History of Science, Charles University in Prague, Prague, Czech Republic; 2 Department of Psychology, Boğaziçi University, Istanbul, Turkey; University of Wroclaw, POLAND

## Abstract

Research on the perception of faces typically assumes that there are some universal values of attractiveness which are shared across individuals and cultures. The perception of attractiveness may, however, vary across cultures due to local differences in both facial morphology and standards of beauty. To examine cross-cultural consensus in the ratings of attractiveness, we presented a set of 120 non-manipulated photographs of Czech faces to ten samples of raters from both European (Czech Republic, Estonia, Sweden, Romania, Turkey, Portugal) and non-European countries (Brazil, India, Cameroon, Namibia). We examined the relative contribution of three facial markers (sexual shape dimorphism, averageness, fluctuating asymmetry) to the perception of attractiveness as well as the possible influence of eye color, which is a locally specific trait. In general, we found that both male and female faces which were closer to the average and more feminine in shape were regarded as more attractive, while fluctuating asymmetry had no effect. Despite a high cross-cultural consensus on attractiveness standards, significant differences in the perception of attractiveness seem to be related to the level of socio-economic development (as measured by the Human Development Index, HDI). Attractiveness ratings by raters from low-HDI countries (India, Cameroon, Namibia) converged less with ratings from Czech Republic than ratings from high-HDI countries (European countries and Brazil). With respect to eye color, some local patterns emerged which we discuss as a consequence of negative frequency-dependent selection.

## Introduction

In social interactions, human attention is rapidly and strongly oriented toward the rich and complex content of human faces. Mere exposure to a face, even one neutral in its expression, can provide information regarding the health condition, age, sex prototypicality, ethnicity, personality, dominance, prestige, trustworthiness, or attractiveness of its bearer [1–5]. Moreover, facial attractiveness conveys information regarding reproductive potential of prospective mating partners [3, 6, 7].

The evolutionary perspective of facial perception assumes that universally shared values of attractiveness exist across individuals and cultures [8, 9]. Because certain individual features such as coloration and symmetry convey valuable genetic information, they are perceived as attractive even in many non-human species [10, 11]. These concepts of attractiveness generally contrast with the maxim ‘beauty is in the eye of the beholder’ [7]. Nonetheless, people do not entirely agree in their assessments of facial attractiveness [12–14]. Agreement in the perception of attractiveness is greater within a single culture than between cultures [15, 16] and some studies have shown that the perception of attractiveness varies across cultures depending on the socio-cultural environment [13, 16–18].

Attractiveness assessments have an impact on an individual’s reproductive success as well as other aspects of social interactions [9, 19, 20]. Facial attractiveness may serve as an indicator of actual health or overall phenotypic condition. The most commonly studied traits involved in judgements of facial attractiveness are sexual shape dimorphism, facial averageness, and symmetry [3, 7, 21]. Below, we briefly review evidence pertaining to these target traits as well as examine the influence of eye color on the perception of facial attractiveness.

### Sexual shape dimorphism (SShD)

Sex-typical facial features are influenced by sex hormones and might thus affect the perception of masculinity, femininity, and also attractiveness. Whereas higher femininity in female faces, interpreted as a signal of fertility [22], is reported as responsible for higher ratings of attractiveness [15, 23–25], women’s preference for masculinity in male faces exhibits a more complex pattern [6, 26]. Masculine facial traits are interpreted as a signal of phenotypic and genetic quality [7], but see [27]. Facial masculinity may further reflect the dominance and social status, which enhance individual’s mate value [4, 28]. For long-term partnership, however, dominance and other personal characteristics connected with masculinity such as aggressiveness are seen as negative or undesirable [29]. In a specific context, more feminine male faces, on the other hand, are preferred as an honest signal of paternal investment [25]. Male facial masculinity is thus preferred only in some contexts or by some individuals, and reasons underlying such contextual and individual differences are not entirely clear.

### Facial averageness

The ‘average is attractive’ hypothesis was introduced by Langlois and Roggman [30], who found that composite faces are more attractive than majority of the individual faces from which the composites were assembled. Even when controlling for a possible confounding effect of smoothness of skin and facial symmetry of composite faces, averageness still retains its influence on attractiveness [31, 32]. Faces closer to the population mean may be favored by stabilizing selection [30]. Indeed, both averageness [33] and attractiveness [34] positively correlate with heterozygosity in major histocompatibility complex genes responsible for immunocompetence. Moreover, averageness is positively related to health [35] and developmental stability [36]. From this point of view, more average faces reflect the health and greater genetic diversity of face bearers who in turn may be preferred in the mate market as attractive, healthy, and parasite-free individuals [3, 37]. Lee et al. [38] reported a genetic component of facial averageness and a significant phenotypic correlation between facial averageness and attractiveness. Facial averageness was not, however, genetically correlated with attractiveness, which contradicts the assumption that averageness reflects genetic quality [38]. Further challenging the ‘average is attractive’ hypothesis, other studies have shown that while average faces of both sexes are perceived as attractive, they are not viewed as the most attractive, that is, it seems that under certain conditions, the perception of attractiveness is independent of averageness [39, 40]. Previous studies have presented an alternative hypothesis, namely that average face is not attractive, and demonstrated that facial attractiveness can be enhanced by atypical characteristics that include a degree of juvenility and/or sex-typicality [41–44]. Nevertheless, it has also been demonstrated that averageness has a greater effect on the perception of attractiveness than juvenilization does [45].

### Fluctuating asymmetry

Traits which are symmetrical at a population level can be described by their degree of fluctuating asymmetry (FA). It is believed that FA reflects developmental instability of an individual, and therefore also genetic and phenotypic conditions that could influence further reproduction [7]. In human faces, exposure to stress during ontogeny is expressed in higher levels of FA [46, 47]. High levels of FA have been linked to various somatic and mental disorders [11], low intelligence [48], and lower health assessment [49]. Studies which used both photographs of real faces and manipulated faces have shown a positive correlation between symmetry and rated attractiveness, e.g., [50]. Some other studies, however, found no such a correlation [51, 52]. Another study [53] found that FA was not an important factor in long-term mating preferences and some scholars believe that the evolutionary importance of FA in determining human attractiveness has been overstated [54]. The hypothesis, that FA honestly signals an individual’s genetic quality, is also criticized based on the argument that many studies supporting this hypothesis used inappropriate statistical methodologies often resulting in overestimated effect sizes [55]. It should also be noted that experiments with manipulated faces may well have yielded varied outcomes largely due to the nature of artificial manipulation [3]. Further research with faces that naturally vary in terms of FA may therefore shed more light on whether and to what extent FA plays a role in attractiveness judgments.

### Eye color

Independently of shape proportions, the coloration of human face is a trait that offers an entirely different type of variability. Whereas the influence of skin texture and color on attractiveness judgments is discussed elsewhere, e.g., [21, 56–58], in our study we focus on eye color, a feature variable mainly in European populations. Unlike the factors presented above, eye color does not seem to have any association with an individual’s fitness [59]. Notwithstanding changes in the brightness of coloration caused by ageing and health condition, eye (as well as hair) color have been considered ‘neutral features’, unlikely to reflect mate quality [59]. According to Edwards et al. [60], iris coloration might be the result of pleiotropic effect associated with selection on pigmentation genes primarily engaged in determining skin or hair color, but not iris coloration. It has been hypothesized that not only natural selection but also sexual selection contributed to recent variations of skin, hair, and eye color [61–63]. A negative frequency-dependent selection in mate choice [64, 65] is a prerequisite for a model introduced by Frost [61, 62] which offers an explanation of the geographical distribution of various eye and hair colors. Frost [61, 62] assumes that ‘rare-color advantage’ of individuals with blue eyes and fair hair could have arisen only in special environmental conditions, a singularity among the many environments which modern humans entered while spreading out from Africa during the Paleolithic.

In comparison with studies on human hair color [65–70], relatively little attention has been paid to eye color’s role in sexual selection. Along with hair color, eye color is a reliable predictor in assortative mating: with respect to these traits individuals prefer partners who resemble their opposite-sex parents [71–73]. Bovet et al. [59] found preferences for self-resembling mates in eye and hair color. In a Norwegian study, Laeng, Mathisen, and Johnsen [74] presented results which support the paternity assurance hypothesis [75]. In his study, blue-eyed men preferred blue-eyed women because such partners provided males greater assurance of recognizing their own offspring. Nevertheless, further evidence did not support this finding, because recessive features were not preferred by male raters in Finland [76], France [59] or among married couples in Slovakia [77]. Kleisner, Kočnar, Rubešová, and Flegr [78] found no relation between perceived attractiveness and eye color in a Czech sample, but revealed a relationship between eye color and facial morphology responsible for the perception of dominance [78] and trustworthiness [79]. Unlike hair color preferences [80, 81], cross-cultural evidence for eye color preferences is lacking.

### Cross-cultural perspective

Cultural context that potentially influence the perception of facial attractiveness can be described in terms of environmental harshness, pathogen load, income inequality, visual experience, and cultural standards. Much of cross-cultural research assumes that mate preferences are shaped towards sex-typical facial characteristics, i.e. femininity in women and masculinity in men, and that this preference is especially strong in areas with limited resources and high pathogen prevalence (for a review, see [17]). Moore et al. [82] described a relationship between Human Developmental Index and women’s preference for cues to testosterone in male faces, while other researchers reported that pathogen stress predicts regional differences in mate preferences [83–87]. These studies generally show that masculine features in male faces are preferred in regions with a high pathogen stress, harsh environment, or low levels of socio-economic development. In these environments, women appear to value masculinity as a cue for protective qualities and/or immunocompetence, which is of potential benefit to the offspring [88], but cf. [89]. Interestingly, male preference for feminine female faces is less pronounced in countries with harsher environment than in countries with better health conditions, and it has been hypothesized that this the result of strategies aimed at resource-holding potential rather than fecundity [86]. A study of Scott et al. [18], on the other hand, showed that both feminine female faces and masculine male faces were less favored in low-HDI than in high-HDI countries. They suggested that the novel environment of industrialized, high-HDI countries may modify attractiveness preferences due to the specific visual diet of their inhabitants. Nonetheless, a recent study by Dixson, Little, Dixson, and Brooks [90] found no support for the hypothesis that pronounced sex-typical facial traits are preferred either in areas with higher urbanization or in environments with a higher pathogen load.

Preference for facial symmetry was reported in harsher and more pathogenic environments [90]. Based on Hadza and European samples, Little, Apicella, and Marlowe [91] suggested that preferences for symmetry can be derived from different ecological conditions, whereby harsher environments lead to a higher preference of symmetry. Using samples of the same populations, Apicella, Little, and Marlowe [92] have also shown that preferences for facial averageness, though reported cross-culturally, are reinforced by visual experience with one’s own population.

### The present study

In summary, a considerable number of studies on face perception brought to light various evidence to the effect that sexual dimorphism, facial averageness, and symmetry influence human mating preferences and most likely have an adaptive value [3, 7, 9]. However, it has also been shown that attractiveness perception is modified by various internal factors, by perceivers’ visual experience, and by the mating context [3, 93]. While emphasizing the environmental context and visual experience, in the present study we engage in a cross-cultural investigation of the relative importance of four facial characteristics–sexual dimorphism, averageness, fluctuating asymmetry, and eye color–for the perception of attractiveness. A set of Czech faces was rated for attractiveness by participants from the Czech Republic, and five other European and four non-European countries.

Based on previous studies, we hypothesize that for both sexes, raters from all populations would rate faces which are closer to the average and have a lower degree of fluctuating asymmetry as more attractive. Further, we hypothesize that possible differences in ratings between the populations should reflect differences in the socio-economic conditions (assessed as HDI) of the target countries. In other words, we expect that the closer the socio-economic environment of raters’ population is to the environment of population of rated faces, i.e. Czech Republic, the greater should be the ratings’ agreement with Czech raters. Based on existing literature, we also expect that symmetrical faces of both sexes and masculine male faces should be rated as more attractive rather in low-HDI than in high-HDI countries. In accordance with Marcinkowska et al. [86] and Scott et al. [18], we suppose that female facial femininity will be more appreciated in industrialized, high-HDI countries. We also assume that preferences for facial averageness will not be substantially affected by socio-economic development. And finally, according to the hypothesis of negative frequency-dependent selection, the ratio of eye color present in a particular population of raters should influence preferences in favor of a characteristic which represents a minority type in that population. We therefore hypothesize that blue-eyed individuals should be perceived as more attractive in populations with a relatively low frequency of blue eyes, and vice-versa, that brown-eyed individuals’ eyes should be preferred in populations with a relatively low frequency of this phenotype.

## Materials and methods

The research was approved by The Institutional Review Board of Charles University, Faculty of Science. Written informed consent was obtained from all participants involved in our study. The data were analyzed anonymously.

### Acquisition of facial photographs

We used a sample of 120 facial photographs (en face portraits): 60 women (mean age±SD = 20.6± 1.2, range: 18–24) and 60 men (mean age±SD = 21.2±2.5, range: 19–34), equally divided between those who have blue and brown color of irises. Individuals with intermediate eye color and those with green eyes were not included due to their ambiguous eye color and relative rareness of these eye colors in the Czech population.

All photographed participants were students of the Faculty of Science, Charles University in Prague, Czech Republic. Participants were asked in advance to refrain from any facial cosmetics and other face decorations. Photographs were taken using a digital camera Nikon D90 with a 50mm lens (full frame equivalent of 75 mm), studio flash, and a reflection screen. The subjects were seated in front of a white background, 1.5m from the camera, and instructed to adopt a neutral facial expression [94]. All photographs were standardized with respect to eye position and clothing of the photographed subjects was digitally cropped so that only a standard, minimal length of neck was visible.

### The rating of photographs

**Fig 1:**
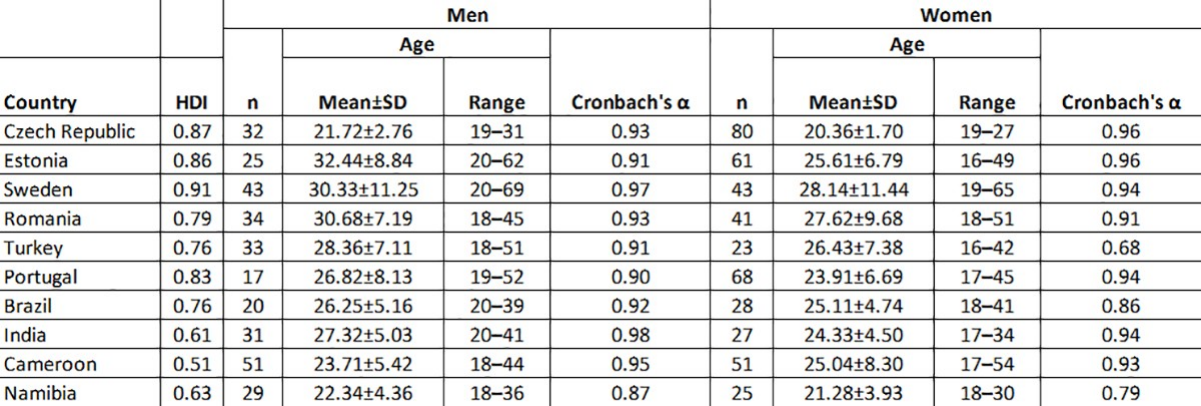
A List of raters according to their country of origin, age distribution, and inter-rater agreement (Cronbach’s α). HDI = Human Development Index.

The set of photographs was rated for attractiveness by volunteers, predominantly university students, in the Czech Republic, Estonia, Sweden, Romania, Turkey, Portugal, Brazil, India, Cameroon, and Namibia. In most cases, data were collected during the year 2014. The Czech participants were recruited from the Charles University in Prague, the Estonian ones from the University of Tartu and Tallinn University, Swedish ones from the Lund University, Romanian ones from the University of Bucharest and West University of Timișoara, Turkish from the Adıyaman University, Portuguese from the Catholic University of Portugal in Braga, Brazilian from the University of São Paulo, Cameroonian from the University of Buea, the Indian sample was drawn from the population of the Sivasagar district in Assam state, and the Namibian sample from suburban sites of the Tseiblaagte and Karasburg communities of the Karas region. For a detailed overview of the demographic characteristics of invited raters, see Fig 1.

Each person rated 60 photographs of faces of the opposite sex on a 7-point, verbally anchored scale, where the lowest number was labelled “very unattractive” and the highest number “very attractive” in the rater’s native language. The sequence of photographs was randomized for each rating session. In countries where daily use of the internet is common, we recruited raters by email invitation and the study was administered online using Qualtrics. Indian, Cameroonian, and Namibian participants were invited personally to a local laboratory and the study was administered offline using the original ImageRater software developed for offline data acquisition. All participants were instructed to rate the photographs in a full screen mode. No time limit was imposed. The rating of all photographs assessed by each rater was converted to Z-scores to eliminate the influence of individual differences in scale use between raters, and perceived attractiveness was calculated for each photograph as its average Z-score across raters of the same sex from the same country.

### Geometric morphometrics

We defined 72 landmarks on each portrait photograph so as to capture the variation in facial shape. To make the description of facial morphology sensitive to curves and locations between true landmarks, we specified 36 sliding landmarks (semilandmarks) from the total of 72 landmarks on each photograph (for definitions of landmark and semilandmark locations on the human face, see [95]). The whole set of faces were landmarked twice to capture information about measurement error for purposes of fluctuating asymmetry quantification. All configurations of landmarks and semilandmarks were superimposed by Generalized Procrustes Analysis (GPA) using the gpagen function included in the geomorph package in R [96]. Positions of semilandmarks were optimized along the tangent directions of facial curves based on minimizing Procrustes distances. Facial averageness was computed as the Procrustes distance between the consensus and each configuration in the set. As a result, the shorter the distance of a face from consensus, the more average the face, whereby lower values indicate higher levels of averageness.

To numerically express the degree of individual expression of facial traits responsible for sexual shape dimorphism, we first pooled the shape coordinates for male and female facial configurations and ran a GPA analysis on these joined male and female coordinates. Then we calculated the position of each individual facial shape along the axis of male–female mean shapes by projecting individual faces onto a vector connecting the male and female consensus [97].

We calculated scores of fluctuating asymmetry using Procrustes ANOVA within MorphoJ, version 1.06d. Facial coordinates of the original and mirrored landmark coordinates (reflected along vertical axis and relabeled) were used as the dependent variable [98, 99]. Independent variables include the main effect of “individuals” (variation among individuals corrected for any effect of asymmetry), the main effect of sides that corresponds to the average difference between the left and right side of the face (directional symmetry), and interaction term of these main effects. Fluctuating asymmetry is quantified as an interaction between the main effects of “individuals” and “sides”. Measurement error was assessed from variations between replicate measurements [100]. Higher FA scores indicate higher facial fluctuating asymmetry.

### Human development index

To approximate cultural differences between the populations of raters, we used the Human Development Index as an appropriate characteristic of each of these populations [18, 82]. HDI scores were extracted from United Nations Development Programme webpage [101], whereby HDI is used to categorize countries by their standard of living as a composite score from 0 to 1 (1 = highest standard of living) calculated from measures of longevity, education, and income.

### Eye color distribution

To compare possible eye color preferences between the populations involved in our study, we first had to establish the relative representation of eye colors in each target population. Since literature on eye color distribution either does not cover the populations we used in our study [102] or is outdated (see the maps based on old and ambiguous data in [103], or [61]), we asked the participants to self-report their own eye color. The data were compiled from a broader set of questionnaires that was based on a larger number of participants than those who participated in the current research. To approximate the eye color distribution in each population, participants were asked to select the category which best corresponds to their own eye color: black-brown, green, grey-blue, or other (see the structure of data in Table 1). Estimated variation is in line with both existing older sources [61, 104] and the European Eye Study [105], which indicates a gradual increase in the frequency of blue-eyed individuals and decrease in those with brown eyes from southern to northern Europe [102].

**Table 1:** Proportional eye color distribution among raters (%).

Country	n	Black–Brown	Green	Grey–Blue	Other^a^
Czech Republic	377	38.5	22.0	39.5	—
*Men*	277	33.0	21.0	46.0	—
*Women*	100	40.4	22.4	37.2	—
Estonia	282	14.5	21.3	56.0	8.2
*Men*	186	19.8	10.4	60.4	9.4
*Women*	96	11.8	26.9	53.8	7.5
Sweden	134	19.4	12.7	50.0	17.9
*Men*	73	16.4	13.1	50.8	19.7
*Women*	61	21.9	12.3	49.3	16.4
Romania	185	58.9	21.1	20.0	—
*Men*	108	57.1	22.1	20.8	—
*Women*	77	60.2	20.4	19.4	—
Turkey	127	85.0	11.0	3.9	—
*Men*	57	87.1	11.4	1.4	—
*Women*	70	82.5	10.5	7.0	—
Portugal	85	84.7	10.6	4.7	—
*Men*	68	64.7	29.4	5.9	—
*Women*	17	89.7	5.9	4.4	—
Brazil	48	75.0	16.7	8.3	—
*Men*	28	70.0	25.0	5.0	—
*Women*	20	78.6	10.7	10.7	—
India	79	97.5	1.3	1.3	—
*Men*	37	97.6	0	2.4	—
*Women*	42	97.3	2.7	0	—
Cameroon	201	100	0	0	—
*Men*	100	100	0	0	—
*Women*	101	100	0	0	—
Namibia	54	100	0	0	—
*Men*	29	100	0	0	—
*Women*	25	100	0	0	—

Absolute numbers of raters were obtained also from other questionnaires.

^a^ The category "other" was included only in questionnaires for Estonian and Swedish raters.

### Statistics

To assess inter-rater reliability, we computed Cronbach’s alpha for each population. Pearson product-moment correlation coefficient was used to explore relationships between all variables. Using Multiple Linear Regression implemented in SPSS 21, we ran 10 separate analyses per sex of the rated faces, one for each population, whereby the mean Z-score of rated attractiveness was used as the dependent variable and measured averageness, SShD, FA, age, and eye color of targets as the predictors. Ratings from the Czech Republic were used as a standard for attractiveness of Czech faces. Pearson’s correlations between attractiveness ratings from the Czech Republic (i.e., the country of origin of individuals whose photographs were rated) and ratings obtained in the other target countries were used for a subsequent Kendall correlation with HDI.

Additionally, we ran complementary analysis on the level of individual ratings with linear mixed-effect models using the “lmer” function within the “lmerTest” R package [106]. Attractiveness ratings were specified as a response variable and age, averageness, FA, SShD, eye color, and HDI as the independent variables. Rater and participant (face) identities were used as random intercepts. The separate models were built for men and women.

## Results

**Fig 2:**
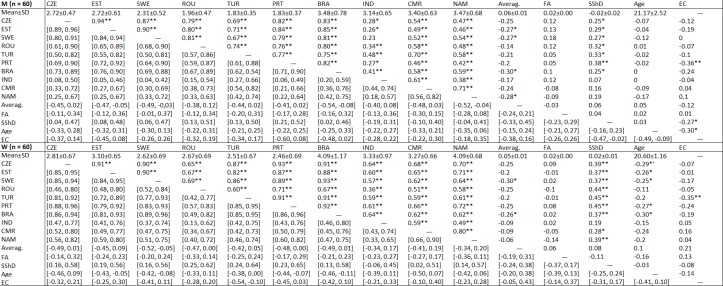
Pearson's correlations between perceived attractiveness judged by opposite-sex raters and physical traits. Confidence intervals are displayed in lower part (CI level = 95%). M = male photos; W = female photos; CZE = Czech Republic; EST = Estonia; SWE = Sweden; ROU = Romania; TUR = Turkey; PRT = Portugal; BRA = Brazil; IND = India; CMR = Cameroon; NAM = Namibia; Averag. = Averageness; FA = Fluctuating Asymmetry; SShD = Sexual Shape Dimorphism; EC = Eye color; Significance levels: * p < 0.05; ** p < 0.01.

Cronbach’s alpha was high for most groups of raters (αs > 0.90). Lower values were recorded for male raters from Namibia (α = 0.87), female raters from Turkey (α = 0.68), and for Brazilian (α = 0.86) and Namibian raters (α = 0.79). For more details, see Fig 1. Descriptive values of all variables as well as Pearson’s correlations between rated attractiveness and physical measurements are listed in Fig 2. Correlation values between countries were obtained from correlations between the average attractiveness score given to individual photographs in one target country and the same score from another target country. Attractiveness ratings both for male and female photographs were relatively constant across all populations. See an overview of all correlations in Fig 2.

### Factors related to perception of attractiveness

To examine the contribution of facial characteristics to attractiveness ratings, we built a linear model with perceived attractiveness as the dependent variable and physical measurements as multiple predictors (see Table 2). Faces closer to the average, both male and female, tended to be regarded as more attractive in a majority of the sampled populations; a significant finding in 6 out of 10 cultures. A similar pattern based on sexual shape dimorphism indicated a relationship between attractiveness and facial femininity: more feminine female faces were rated as more attractive by respective opposite-sex raters. This was a significant finding in all cultures except India. In majority of high-HDI countries, namely in Estonia, Sweden, Romania, Turkey, and Portugal, female raters preferred also more feminine faces in men. In some cultures, attractiveness ratings were also influenced by targets’ age, whereby younger female faces were perceived as more attractive. This was a significant finding in the Czech Republic, Estonia, Portugal, and Brazil. In Cameroon, Namibia, and India, neither facial averageness nor age significantly influenced the attractiveness ratings of female photographs. Indian raters of both sexes seem exceptional in the sense that their attractiveness ratings did not reveal any significant importance of averageness, age, or even SShD. Further, we found no effect of fluctuating asymmetry on attractiveness ratings in any of the rater populations.

**Table 2:** Relationship between rated attractiveness and variables measured by multiple regression.

	Men					Women				
Predictors per country	Full model	B	SE	t-value	p-value	Full model	B	SE	t-value	p-value
Czech Republic	F = 2.071					F = 4.658				
	p = 0.083					p = **0.001**				
	R^2^ = 0.161					R^2^ = 0.301				
*Averageness*		-11.351	5.307	-2.139	**0.037**		-15.911	7.195	-2.211	**0.031**
*SShD*		8.070	4.419	1.826	0.073		19.220	5.436	3.536	**0.001**
*FA*		11.443	13.136	0.871	0.388		14.042	14.649	0.959	0.342
*Age*		-0.021	0.027	-0.760	0.450		-0.119	0.060	-1.989	0.052
*Eye Color*		-0.122	0.143	-0.855	0.396		-0.035	0.140	-0.247	0.806
Estonia	F = 2.808					F = 4.415				
	p = **0.025**					p = **0.002**				
	R^2^ = 0.206					R^2^ = 0.290				
*Averageness*		-12.978	5.340	-2.430	**0.018**		-14.969	7.882	-1.899	0.063
*SShD*		8.999	4.447	2.024	**0.048**		19.783	5.955	3.322	**0.002**
*FA*		12.421	12.220	0.940	0.352		4.961	16.047	0.309	0.758
*Age*		-0.020	0.027	-0.718	0.476		-0.159	0.066	-2.423	**0.019**
*Eye Color*		-0.199	0.144	-1.377	0.174		0.089	0.154	0.580	0.564
Sweden	F = 2.534					F = 4.729				
	p = **0.039**					p = **0.001**				
	R^2^ = 0.190					R^2^ = 0.305				
*Averageness*		-11.906	5.420	-2.197	**0.032**		-18.923	7.917	-2.390	**0.020**
*SShD*		10.037	4.513	2.224	**0.030**		19.909	5.981	3.329	**0.002**
*FA*		17.767	13.416	1.324	0.191		7.842	16.118	0.487	0.629
*Age*		-0.024	0.028	-0.874	0.386		-0.126	0.066	-1.905	0.062
*Eye Color*		0.015	0.146	0.100	0.921		-0.158	0.154	-1.025	0.310
Romania	F = 1.752					F = 4.314				
	p = 0.139					p = **0.002**				
	R^2^ = 0.140					R^2^ = 0.282				
*Averageness*		-5.933	4.840	-1.226	0.226		-5.201	2.182	-2.383	**0.021**
*SShD*		9.977	4.031	2.475	**0.016**		6.517	1.649	3.953	**<0.001**
*FA*		9.962	11.982	0.831	0.409		-1.699	4.443	-0.382	0.704
*Age*		0.000	0.025	0.015	0.988		-0.010	0.018	-0.553	0.582
*Eye Color*		-0.002	0.131	-0.017	0.987		0.014	0.043	0.317	0.752
Turkey	F = 2.090					F = 6.724				
	p = 0.081					p < **0.001**				
	R^2^ = 0.162					R^2^ = 0.384				
*Averageness*		-6.521	3.591	-1.816	0.075		-8.511	6.355	-1.339	0.186
*SShD*		7.621	2.991	2.548	**0.014**		19.271	4.801	4.014	**<0.001**
*FA*		2.032	8.891	0.229	0.820		6.353	12.938	0.491	0.625
*Age*		-0.005	0.018	-0.280	0.781		-0.102	0.053	-1.936	0.058
*Eye Color*		-0.031	0.097	-0.321	0.749		-0.362	0.124	-2.919	**0.005**
Portugal	F = 4.349					F = 6.782				
	p = **0.002**					p < **0.001**				
	R^2^ = 0.287					R^2^ = 0.386				
*Averageness*		-9.462	4.398	-2.151	**0.036**		-15.252	7.393	-2.063	**0.044**
*SShD*		9.168	3.663	2.503	**0.015**		23.481	5.585	4.204	**<0.001**
*FA*		3.566	10.887	0.327	0.745		17.974	15.052	1.194	0.238
*Age*		-0.022	0.023	-0.998	0.323		-0.135	0.062	-2.195	**0.033**
*Eye Color*		-0.321	0.119	-2.700	**0.009**		-0.268	0.144	-1.856	0.069
Brazil	F = 3.064					F = 4.857				
	p = **0.017**					p = **0.001**				
	R^2^ = 0.221					R^2^ = 0.310				
*Averageness*		-11.969	4.292	-2.789	**0.007**		-16.109	8.122	-1.983	0.052
*SShD*		5.873	3.574	1.643	0.106		19.832	6.136	3.232	**0.002**
*FA*		6.707	10.624	0.631	0.531		7.275	16.537	0.440	0.662
*Age*		-0.011	0.022	-0.509	0.613		-0.161	0.068	-2.382	**0.021**
*Eye Color*		-0.218	0.116	-1.882	0.065		-0.209	0.158	-1.319	0.193
India	F = 0.590					F = 0.808				
	p = 0.708					p = 0.549				
	R^2^ = 0.052					R^2^ = 0.070				
*Averageness*		-9.620	7.179	-1.340	0.186		-8.820	11.291	-0.781	0.438
*SShD*		2.806	5.979	0.469	0.641		12.769	8.531	1.497	0.140
*FA*		15.626	17.772	0.879	0.383		3.525	22.989	0.153	0.879
*Age*		-0.002	0.037	-0.048	0.962		-0.086	0.094	-0.910	0.367
*Eye Color*		-0.061	0.194	-0.313	0.756		0.111	0.220	0.503	0.617
Cameroon	F = 1.269					F = 2.309				
	p = 0.291					p = 0.057				
	R^2^ = 0.105					R^2^ = 0.176				
*Averageness*		-8.475	4.483	-1.890	0.064		-7.589	7.352	-1.032	0.307
*SShD*		5.420	3.734	1.452	0.152		12.908	5.554	2.324	**0.024**
*FA*		-7.986	11.098	-0.720	0.475		-8.310	14.962	-0.555	0.581
*Age*		-0.011	0.023	-0.500	0.619		-0.095	0.061	-1.553	0.126
*Eye Color*		0.035	0.121	0.289	0.773		0.215	0.143	1.497	0.140
Namibia	F = 2.212					F = 2.833				
	p = 0.066					p = **0.024**				
	R^2^ = 0.170					R^2^ = 0.208				
*Averageness*		-9.700	4.287	-2.263	**0.028**		-3.949	6.167	-0.640	0.525
*SShD*		6.800	3.578	1.905	0.062		14.288	4.659	3.067	**0.003**
*FA*		-8.935	10.613	-0.842	0.404		-13.360	12.557	-1.064	0.292
*Age*		-0.023	0.022	-1.029	0.308		-0.077	0.051	-1.492	0.142
*Eye Color*		0.082	0.116	0.713	0.479		0.069	0.120	0.575	0.568

Results which reached the level of significance (p<0.05) are in boldface. Correlation of perceived attractiveness with SShD of women perceived by Czech, Estonian, Swedish, Romanian, Turkish, Portuguese, Brazilian, and Namibian male raters, and correlation with eye color of women perceived by Turkish male raters remained statistically significant (p<0.05) after Bonferroni correction. SShD = Sexual Shape Dimorphism; FA = Fluctuating Asymmetry.

The eye color of targets had a limited impact on attractiveness ratings. Blue-eyed men were perceived as more attractive than brown-eyed men by female Portuguese raters (r = -0.36, n = 60, p < 0.01, 95% CI [-0.60, 0.08]). Blue-eyed women were significantly preferred as more attractive by male Turkish raters (r = -0.35, n = 60, p < 0.01, 95% CI [-0.54, -0.10]).

### Cross-cultural agreement in the perception of attractiveness

**Fig 3:**
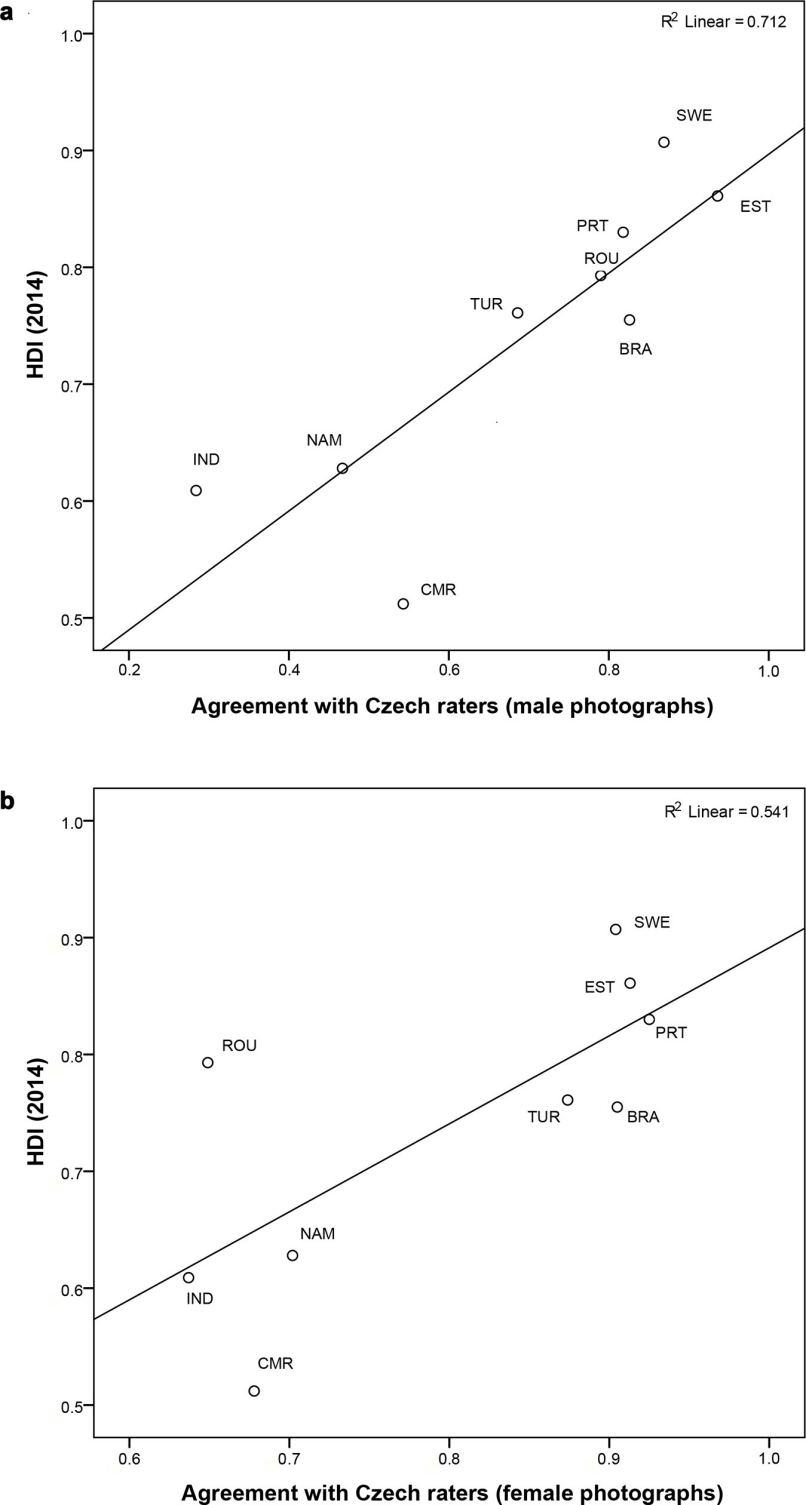
Relationship between the Human Development Index (HDI) and Agreement with Czech Raters. Using Kendall correlation, we identified a significant relationship for (a) male (τ = 0.67, n = 9, p = 0.01, 95% CI [0.25, 1]) but not (b) female faces (τ = 0.44, n = 9, p = 0.10, 95% CI [-0.10, 0.86]). On x-axis, agreement with Czech raters is expressed by values of bivariate correlations between Czech ratings and ratings of each target country.

To explain the pattern of correlations among different countries, we examined the relationship between HDI and facial attractiveness. We computed Kendall correlations between the HDI and values of bivariate correlations between the Czech ratings and ratings of each target country. Fig 3 shows a significant relationship for male faces (τ = 0.67, n = 9, p = 0.01, 95% CI [0.25, 1]), but not female ones (τ = 0.44, n = 9, p = 0.10, 95% CI [-0.10, 0.86]). Graphs for both sexes, however, indicate that participants in low-HDI countries disagree with Czech raters more, whereas European and Brazilian participants, i.e. raters from countries with HDI scores closer to the Czech Republic (HDI = 0.87), do converge with the Czech ratings. Only Romanian male raters are an exception: their ratings were in a relatively low agreement with Czech raters (r = 0.68, n = 60, p < 0.01, 95% CI [0.46, 0.80]).

Linear mixed-effect modelling corroborated the general pattern of results from regression analyses based on averages of attractiveness ratings. Detailed results for mixed-effect models are summarized in Table 3.

**Table 3:** Summary of the results of linear mixed-effects modeling.

**Men**	**Random effects**	**Variance**	**SD**		
	Rater’s identity	0.563	0.751		
	Face’s identity	0.177	0.420		
	**Fixed effects**	**Estimate**	**SE**	**t-value**	**p-value**
	Intercept	3.898	0.658	5.923	<0.001***
	Averageness	-11.096	4.443	-2.498	0.016*
	SShD	8.384	3.700	2.266	0.028*
	FA	8.072	10.997	0.734	0.466
	Age	-0.017	0.023	-0.744	0.460
	Eye Color	-0.110	0.120	-0.919	0.362
	Estonia	-0.057	0.130	-0.443	0.658
	Sweden	-0.470	0.144	-3.260	0.001**
	Romania	-0.821	0.145	-5.653	<0.001***
	Turkey	-0.947	0.180	-5.249	<0.001***
	Portugal	-0.954	0.126	-7.584	<0.001***
	Brazil	-0.129	0.167	-0.773	0.440
	India	0.362	0.170	2.130	0.034*
	Cameroon	0.618	0.137	4.522	<0.001***
	Namibia	0.689	0.175	3.942	<0.001***
**Women**	**Random effects**	**Variance**	**SD**		
	Rater’s identity	0.788	0.888		
	Face’s identity	0.308	0.555		
	**Fixed effects**	**Estimate**	**SE**	**t-value**	**p-value**
	Intercept	5.748	1.444	3.981	<0.001***
	Averageness	-14.928	7.739	-1.929	0.059
	SShD	20.135	5.847	3.444	0.001**
	FA	3.949	15.756	0.251	0.803
	Age	-0.121	0.064	-1.885	0.065
	Eye Color	-0.039	0.151	-0.260	0.796
	Estonia	0.292	0.240	1.217	0.225
	Sweden	-0.189	0.210	-0.902	0.368
	Romania	-0.144	0.221	-0.649	0.517
	Turkey	-0.299	0.223	-1.340	0.181
	Portugal	-0.350	0.270	-1.298	0.195
	Brazil	0.250	0.256	0.976	0.330
	India	0.519	0.226	2.291	0.023*
	Cameroon	0.465	0.203	2.294	0.022*
	Namibia	1.279	0.230	5.554	<0.001***

SShD = Sexual Shape Dimorphism; FA = Fluctuating Asymmetry

Significance levels

*p <0.05

**p<0.01

***p<0.001

## Discussion

In our study, we offer further support for the hypothesis that although certain features of the human face are perceived as attractive across cultures, this perception is variable. Our findings indicate that this variability is related to environmental and socio-cultural factors. We found significant correlations between two facial shape traits–sexual shape dimorphism and averageness–and perceived attractiveness. With respect to the third examined trait, fluctuating asymmetry, we found no relation to rated attractiveness in any of the target populations. We also found that eye color seems to be a culture-specific cue to the perception of attractiveness. Findings for each of these facial traits are discussed below.

### Sexual shape dimorphism

More feminine female faces were perceived as more attractive in all populations except for India. In men, facial attractiveness was also influenced by sex-typicality in favor of feminized rather than masculinized faces. Note, however, that this was a significant effect only in Sweden, Estonia, Romania, Turkey, and Portugal. This indicates that the perception of an attractive face is influenced more by sex-typical traits than by averageness. Using a sample of non-manipulated faces, we have demonstrated a cross-cultural validity of findings of Perrett et al. [25] who reported agreement in preference for feminized female faces in both Japanese and European perceivers. Unlike in Perrett et al. [25], our results do not run counter the averageness hypothesis but rather show that female attractiveness is driven by both sex-typicality and averageness. Nevertheless, feminized rather than average faces in women were preferred in a larger group of countries (see results for Turkey, Brazil, and Namibia).

None of the cultures we studied exhibited a preference for masculinized male faces. Quite the contrary, feminized male faces were preferred by women in most European populations. Equivocal role of sexual dimorphism in male facial attractiveness has been interpreted as a consequence of female tradeoff strategies [25, 83, 107]. Preferences for feminized facial shapes in men may be motivated by prospective partner’s characteristics such paternal skills, cooperativeness, and trustworthiness [25]. These characteristics may compensate for preference for those masculine facial traits which are believed to be cues to dominance [108], or aggressiveness and competitiveness [109], in short, for traits associated with ease of access to resources and ability to protect mate and offspring [110]. It has been also shown that women who control their own resources may prefer men who are ‘helpers in the nest’ over masculine men who promise the benefit of good genes [111]. One limitation of our study is that we did not ask our raters about their relationship status, because this factor might further modulate the effects of environmental conditions on women’s preferences for facial masculinity as reported in Lyons, Marcinkowska, Moisey, and Harrison [112]. (See also further discussion on preferences for SShD from a cross-cultural perspective below.)

Alternatively, absence of preference for masculinity in male faces could be examined from the perspective of conflicting preferences for relatively feminine shape but relatively masculine skin color. This model was theoretically proposed by Said and Todorov [40] and experimentally tested using composite faces by Carrito et al. [113], who found a preference for feminine-shaped European male faces and a preference for masculinization in the color component relative to the shape component of male faces. We did not examine the perception of skin coloration in our study and due to low variation of skin color in Czech, and indeed most European populations [58], it is not certain that in our sample of non-manipulated photographs these two aspects could be differentiated.

### Facial averageness

Facial averageness was generally perceived as attractive: the correlation was significant in about one half of the target populations, predominantly the European ones. Considering that only Czech faces were rated, these results may indicate that non-European raters are not sensitive to a prototypical European face since their visual experience is guided by prototypic standards which are based on their own population. This seems to partly contradict the results of studies by Rhodes et al. [32, 35] who found that faces manipulated to appear closer to the average are rated as more attractive irrespective of ethnicity of either the targets or the raters. In the latter study, Asian perceivers did not prefer own-ethnicity averaged composites over other-ethnicity or mixed ethnicity composites [35]. Rhodes, Jeffery, Watson, Clifford, and Nakayama [114] suggest that a process of perceptual adaptation can rapidly adjust raters’ preferences to fit the rated faces and thus re-set an average prototypical face. It is, however, questionable to what degree these mechanisms are involved when raters are confronted with an unusual population of faces [88]. Our results from non-European raters may indicate that raters cannot recalibrate their prototype of averageness when exposed to other-ethnicity photographs for just a short time during evaluation.

### Fluctuating asymmetry

In our results, the degree of FA in faces of either sex did not seem to be linked to the perception of attractiveness. This contrasts with several previous studies [32, 50, 115–117]. Nevertheless, it should be noted that research on the relation between facial symmetry and perceived attractiveness has been yielding inconsistent results, as documented in a considerable number of reports of negative results [51, 52, 118, 119], or in meta-analyses by Rhodes and Simmons [55], Van Dongen [54, 120], and Van Dongen and Gangestad [121]. Rhodes and Simmons [55] reported moderate effect of facial FA on attractiveness but found little evidence for a hypothesis that FA signals mate quality. In a recent study, facial averageness yielded a large effect whereas FA yielded a small effect on attractiveness [122]. In a study of Mogilski and Welling [123], potential mate’s facial sexual dimorphism was prioritized over facial symmetry. In our study, we used a similar sample of non-manipulated photographs (120 compared to 200 subjects, in both studies the subjects were students from European universities) and used same method of FA computation as Van Dongen [119] who found no association between FA and facial attractiveness. Our results are also in line with Kleisner et al. [58] who found no relation between FA and rated attractiveness in two samples of African faces rated across three populations. Moreover, Graham and Özener [124] in their thorough review on fluctuating asymmetry in humans questioned the importance of FA as an honest indicator of fitness and suggested that research should rather focus on examining the relation of FA to directional asymmetry which correlates with the individual’s low developmental stability.

### Eye color

With respect to the influence of eye color on the perception of attractiveness, we observed a pattern explicable by a negative frequency-dependent selection. Specifically, we found preference for blue eyes in Turkey and Portugal where the trait is not common. In contrast, however, we found no similar preference for the less common brown eyes in Estonia or Sweden. Our data also show a notable difference in preference for blue eyes between the sexes. While in Portugal and in Brazil, blue-eyed men–but not women–were preferred as more attractive, in Turkey, blue-eyed women–but not men–were rated as more attractive. This could be interpreted from the perspective of the social status of women in a given society. Given that the Turkish society is characterized by a relatively high degree of gender inequality [125–127], one could speculate that the position of Turkish women in courting is rather passive, which is in turn reflected in a more conservative rating of facial attractiveness. In other words, it is possible that unlike their Portuguese counterparts, Turkish women do not pay attention to special and ‘redundant’ traits such as eye color. Or, from the perspective of Turkish men, the traditional structure of Turkish society allows only men to take initiative in courtship and this may in turn influence their preferences. Karandashev et al. [128] reported that eyes play a role in romantic courtship among Georgian, Portuguese, French, but not Russian respondents, yet only Georgian men, not women, focused their attention more on eyes than any other facial feature. Taking into account that Georgia is a geographical neighbor to Turkey, it is possible that these cultures, however distinct in religious beliefs, share a similar view on the importance of eyes in the perceived attractiveness of women. This preference may have been strengthened by a Muslim tradition of female face covering in the Middle East which leaves only the eyes and their surrounding visible and available for non-verbal facial communication [128].

Further, the attractiveness preferences of Turkish population might be significantly shaped by relatively long period of cultural and political interconnectedness with East-Central European region. Beside the well-documented genetic impact of Ottoman occupation on ethnic groups of East-Central Europe [129], the admixture with Slavic genes has taken part in the very center of the empire–a royal harem [130]. In recent times, Russian immigration to Turkey may enrich the mate market [131,132] with rare, and thus desirable phenotypes, including blue eyes. Preferences for atypical appearance of women are also reflected in popular folk songs of the region such as *Sarı Gelin*, i.e. blond-haired bride [133].

Another factor related to perception of eyes in Turkey is the concept of evil eye (*nazar*), a still-present superstition with different additional cultural layers ascribed to otherwise ancient meanings [134]. A widespread amulet in Middle-East, an evil eye bead, has the shape and color of a blue eye. Blue eye color might be assigned a special meaning because in a predominantly brown-eyed society, blue eyes are uncommon, strange, and therefore perceived as potentially dangerous. Alternatively, blue eye color may be valued because the highest deity in old Turkic religions resides in the blue sky. The recent meaning of the blue eye amulet, popular in all segments of society, may be based either on its original protective role, whereby it is viewed as an expression of good luck and greetings, or just on an aesthetic function [134]. It is therefore possible that both Turkish men and women unconsciously attach different importance to the same facial feature.

Alternatively, preferences for eye colors may be driven by repeated exposure. The more one is exposed to a particular trait (e.g., eye color), the greater should be the positive evaluation of that trait [72, 80, 135]. On a population level, we did not find that the prevailing eye color is more preferred than a rare one in any of the target countries. On the other hand, one cannot draw conclusions based solely on the ratio of eye colors in populations. To remove these limitations, we should have also asked the raters about their parents’ and partner’s eye colors. That would at least approximately determine the environment in which the participants have been brought up and currently live.

### Cross-cultural agreement and differences

Despite a generally high agreement in attractiveness ratings between cultures, which has been reported in other studies [43, 136], we have also observed a prominent pattern in correlations which might reflect differences in the HDI of participating populations, see [18, 82]. Most notable is the gap between two clusters, one consisting of European countries plus Brazil, the other of other non-European populations. The disparity between European and non-European populations is parallel to a greater agreement on facial attractiveness perception within than between populations [25, 109, 137, 138]. As pointed out by Sorokowski et al. [138], criteria of attractiveness may vary between cultures due to the ecological conditions of a given population, but all populations substantially agree on unattractiveness, which is according to this study a better proxy of health and biological quality. If there is a common basis for agreement on what is not attractive, or, to express it less crudely, if the perception of attractiveness is part of our evolutionary heritage [139], can we at least partly identify the source of culture-specific tastes?

Our data indicate that we may see fundamental differences in the perception of attractiveness due to (1) the degree of divergence in ecological conditions approximated by the level of socio-economic development, and due to (2) familiarity with the population to which the preferences are attached. It is well known that socio-economic development influences the perception of attractiveness of human bodily morphology [140–143]. In a review dedicated to the perception of body size, Swami [143] argued that despite a large degree of uniformity in body size ideals due to Westernization [140], the socio-economic status of perceivers does lead to significant differences in preferences.

Whereas the body or its particular features such as muscularity, fat level, waist-to-hip ratio, or height can be directly related to fitness-dependent qualities and it has been reported that their perception is influenced by environmental conditions and moderated by Westernization, the perception of faces is influenced by yet another important component, namely familiarity with facial diversity within a population. It has been shown that familiarity with facial proportions results in a more accurate estimation of body weight in a population of one’s own ethnicity than other ethnicity [144] and may be the cause of differences in preferences between rural and urban populations [145]. Perceived attractiveness of Czech faces in populations like India, Cameroon, or Namibia could thus be influenced by a relative lack of familiarity with European faces. Different experiences may result in different norms of attractiveness and this could not only overshadow sensitivity to specific, unfamiliar traits such as eye color, but also influence the perception of biologically-based traits of attractiveness such as averageness and sexual dimorphism. On the example of chin morphology, Thayer and Dobson [146] documented that geographic differences in chin shape are consistent with population-specific mating preferences that favor a familiar appearance. Additionally, the perception of an ‘unfamiliar’ population may be influenced by cross-race effect, that is, by a more accurate recognition of own-culture than other-culture faces [147].

Although we found correlations between perceived facial attractiveness and the level of socio-economic development, one ought to consider with caution the degree to which one can rely on HDI to explain cross-cultural differences. Our findings are in line with Marcinkowska et al. [86], who used the National Health Index as a proxy for regional differences in men’s attractiveness preferences and found that facial femininity was less favored in countries with worse health conditions. Similarly, Scott et al. [18] found that men’s preferences for feminine female faces are less pronounced in low-HDI countries. On the other hand, we cannot simply infer that masculinity in men is preferred in high-HDI countries [18]. Quite the opposite, preferences for feminine male faces in relatively wealthy regions rather than in harsher environments correspond with earlier findings that masculinity is more valued in less developed regions [82–85, 148]. Nevertheless, an even more complex pattern emerges from the findings of Batres and Perrett [149] who had shown that raters without internet access perceive feminine male faces as more attractive, or Dixson et al. [90] who reported no preference for masculine male faces and feminine female faces neither in regions with high pathogen load nor in areas of urban development. It is therefore evident that rather than relying solely on differences between countries as approximated by HDI, pathogen stress, national income inequality, or other indices, a detailed cross-cultural investigation requires awareness of the sub-structure and cultural specifics of the target regions [150]. Nevertheless, our contribution supports a conclusion that both facial femininity in women and averageness in general do play a decisive role especially in countries with a higher HDI. In this sense, we can agree with the argument proposed by Scott et al. [18] who claimed that the novel environment of Westernized, urban, high-HDI society creates space for new opportunities where a broader scale of attractiveness attributes is taken in consideration. Finally, we should keep in mind that we used a European photoset, which implies that participants from low-HDI countries such as Cameroon, Namibia, and India have naturally less direct contact with facial stimuli of European origin compared to cultures in closer physical proximity to the Czech Republic.

### Limitations and future directions

A cross-cultural comparison would have provided more insights into local cultural specifics had we asked a broader set of questions related to the raters themselves. Factors which influenced the raters’ assessment of attractiveness could be influenced by their marital status, family background, personality traits, sociosexuality, social class, and other additional considerations. For example, due to absence of relevant information about Indian raters it is difficult to figure out why, in case of this particular culture, we found no association between perceived attractiveness and the traits followed by this study. It is then only an uncorroborated assumption to claim that the decisions of Indian men might be moderated by, for instance, social class or traditional familial rules. Their perception of female attractiveness could be influenced by a mixture of various factors involved in mate preferences, such as religiosity (‘religious’ as a preferred trait in women is reported by Basu and Ray [151]). It is also possible that the perception of male attractiveness is influenced by the sexually restricted behavior of Indian women [152]. Further, the participants’ attitudes to traditional marriage practices could also significantly uncover the differences in preferences of collectivistic societies such as India or Turkey [153, 154]. Moreover, information about the eye color of family members and partners of our raters would have helped to answer questions related to assortative mating [72]. Similarly, in order to disentangle the variance in women’s preference for male facial masculinity on a cross-cultural level, one should first of all investigate the various differences that could reflect a tradeoff between costs and benefits, where preferences for a more masculine or more feminine male mate are dependent on the phase of the menstrual cycle [155], partnership status [156], relationship type [157], self-rated attractiveness [158], or the male counterpart’s hormone levels [159].

While a set of non-manipulated photographs has the advantage of reflecting a natural variation in appearance, it also carries a disadvantage because variation in facial features may conceal possible attractiveness-influencing factors that would be more apparent in manipulated images. Moreover, we did not sort our set by hair colors. Different combinations of hair and eye color on the one hand and hair style on the other may have also partly influenced the ratings. Further, some limitations may be due to the fact that we have intentionally reduced eye colors to only two distinct categories of brown and blue. Nevertheless, one fifth of Czech population reports having green eyes. In one study, green-eyed women also reported better health condition than participants with other eye colors [160]. Both due to its rareness and a putative link associating eye color with health, this particular eye color might be considered as most appealing in women. In India, for example, green eyes might be perceived as exceptionally attractive: note, for instance, the Bollywood female star Aishwarya Rai [161]. Alongside other rare traits, green eye color might further play a role in some Asian cultures where local standards of beauty are gradually conforming to international standards of beauty [162].

To assure a reliable cross-cultural comparison that would reflect the differences in HDI, one should consider increasing the number of cultures involved or include subsamples from non-European countries which vary in their degree of Westernization. It is worth noting, for instance, that our Turkish sample was drawn from Adıyaman, a city located in southeastern Turkey, an area that more traditional than most other Turkish regions. Recruiting a sample from northwestern Turkey, which has historically been more open to European influence and is socially more liberal, could produce different results. In sum, in interpreting our results, it should be kept in mind that our samples are not nationally representative.

## Conclusions

Based on the rating of European faces in ten populations, both European and non-European, we found support for the hypothesis that averageness and sexual dimorphism in human face play a significant role for attractiveness assessment, whereas the influence of fluctuating asymmetry is negligible. In line with negative frequency-dependent selection, the blue-eyed phenotype influenced ratings only in those cultures where it is present but not common. And last but not least, we found that factors which influence the perception of facial attractiveness in different populations are affected by the relevant socio-cultural background, here reflected in the HDI index: more convergent socio-cultural background of raters’ population and the population whose faces are rated leads to more similar ratings in these two populations. Explanations of our findings are tentative, and we offer directions for further examination, especially with respect to involving other cultures of both perceivers and, particularly, the rated subjects.

## Supporting information

S1 Table Results of procrustes analysis and attractiveness ratings for each photo. (XLSX)

## References

[1] Henderson AJ, Holzleitner IJ, Talamas SN, Perrett DI. Perception of health from facial cues. Philos Trans R Soc Lond B Biol Sci [Internet]. The Royal Society; 2016 5 5 [cited 2018 Jul 22];371(1693). Available from: http://www.ncbi.nlm.nih.gov/pubmed/27069057 10.1098/rstb.2015.0380 PMC4843618 27069057

[2] Keating CF. Charismatic faces: Social status cues put face appeal in context Facial attrativeness: Evolutionary, cognitive, and social perspectives. Westport, CT, US: Ablex Publishing; 2002 p. 153–92.

[3] Little AC, Jones BC, Debruine LM. Facial attractiveness: Evolutionary based research. Philos Trans R Soc B Biol Sci [Internet]. 2011 6 12 [cited 2017 Feb 2];366(1571):1638–59. Available from: http://www.pubmedcentral.nih.gov/articlerender.fcgi?artid=3130383&tool=pmcentrez&rendertype=abstract 10.1098/rstb.2010.0404 PMC3130383 21536551

[4] Mueller U, Mazur A. Facial dominance in Homo sapiens as honest signalling of male quality. Behav Ecol. 1997;8(5):569–79.

[5] Todorov A. Evaluating faces on trustworthiness: An extension of systems for recognition of emotions signaling approach/avoidance behaviors. Ann N Y Acad Sci. 2008;1124:208–24. 10.1196/annals.1440.012 18400932

[6] Rhodes G. The Evolutionary Psychology of Facial Beauty. Annu Rev Psychol [Internet]. 2006;57(1):199–226. Available from: http://www.annualreviews.org/doi/10.1146/annurev.psych.57.102904.190208 16318594 10.1146/annurev.psych.57.102904.190208

[7] Thornhill R, Gangestad SW. Facial attractiveness. 1999;3(12):452–60. 10.1016/s1364-6613(99)01403-5 10562724

[8] Cunningham MR, Roberts AR, Barbee AP, Druen PB, Wu C-H. “Their ideas of beauty are, on the whole, the same as ours”: Consistency and variability in the cross-cultural perception of female physical attractiveness. J Pers Soc Psychol [Internet]. 1995 [cited 2018 Jul 25];68(2):261–79. Available from: http://doi.apa.org/getdoi.cfm?doi=10.1037/0022-3514.68.2.261

[9] Langlois JH, Kalakanis L, Rubenstein AJ, Larson A, Hallam M, Smoot M. Maxims or myths of beauty? A meta-analytic and theoretical review. Psychol Bull [Internet]. 2000;126(3):390–423. Available from: http://doi.apa.org/getdoi.cfm?doi=10.1037/0033-2909.126.3.390 10825783 10.1037/0033-2909.126.3.390

[10] Andersson MB. Sexual Selection. Princeton University Press 1994.

[11] Møller AP, Thornhill R. Bilateral Symmetry and Sexual Selection: A Meta‐Analysis. Am Nat. 1998; 10.1086/286110 18811416

[12] Bronstad PM, Russell R. Beauty is in the “we” of the beholder: Greater agreement on facial attractiveness among close relations. Perception. 2007;36(11):1674–81. 10.1068/p5793 18265847

[13] Germine L, Russell R, Bronstad PM, Blokland GAM, Smoller JW, Kwok H, et al Individual Aesthetic Preferences for Faces Are Shaped Mostly by Environments, Not Genes. Curr Biol. 2015;25(20):2684–9. 10.1016/j.cub.2015.08.048 26441352 PMC4629915

[14] Hönekopp J. Once more: Is beauty in the eye of the beholder? Relative contributions of private and shared taste to judgments of facial attractiveness. J Exp Psychol Hum Percept Perform. 2006;32(2):199–209. 10.1037/0096-1523.32.2.199 16634665

[15] Jones D, Hill K. Criteria of facial attractiveness in five populations. Hum Nat. 1993;4(3):271–96. 10.1007/BF02692202 24214367

[16] Penton-Voak IS, Jacobson A, Trivers R. Populational differences in attractiveness judgements of male and female faces: Comparing British and Jamaican samples. Evol Hum Behav. 2004;25(6):355–70.

[17] Pisanski K, Feinberg DR. Cross-Cultural Variation in Mate Preferences for Averageness, Symmetry, Body Size, and Masculinity. Cross-Cultural Res [Internet]. 2013;47(2):162–97. Available from: 10.1177/1069397112471806

[18] Scott IM, Clark AP, Josephson SC, Boyette AH, Cuthill IC, Fried RL, et al Human preferences for sexually dimorphic faces may be evolutionarily novel. Proc Natl Acad Sci [Internet]. 2014;111(40):14388–93. Available from: http://www.pnas.org/lookup/doi/10.1073/pnas.1409643111 25246593 10.1073/pnas.1409643111 PMC4210032

[19] Jokela M. Physical attractiveness and reproductive success in humans: Evidence from the late 20 century United States. Evol Hum Behav. 2009;30(5):342–350. 10.1016/j.evolhumbehav.2009.03.006 21151758 PMC3000557

[20] Prokop P, Fedor P. Physical attractiveness influences reproductive success of modern men. Journal of Ethology. 2011;29(3):453–458.

[21] Foo YZ, Simmons LW, Rhodes G. Predictors of facial attractiveness and health in humans. Sci Rep [Internet]. Nature Publishing Group; 2017;7(June 2016):1–12. Available from: 10.1038/s41598-016-0028-x 28155897 PMC5290736

[22] Johnston VS. Female facial beauty: The fertility hypothesis. Pragmat Cogn [Internet]. 2000 5 22 [cited 2018 Jul 24];8(1):107–22. Available from: https://benjamins.com/catalog/pc.8.1.06joh

[23] Grammer K, Thornhill R. Human (Homo sapiens) facial attractiveness and sexual selection: The role of symmetry and averageness. J Comp Psychol [Internet]. 1994 [cited 2018 Jul 24];108(3):233–42. Available from: http://doi.apa.org/getdoi.cfm?doi=10.1037/0735-7036.108.3.233 7924253 10.1037/0735-7036.108.3.233

[24] Little AC, Jones BC, Feinberg DR, Perrett DI. Men’s strategic preferences for femininity in female faces. Br J Psychol [Internet]. Wiley/Blackwell (10.1111); 2014 8 [cited 2018 Jul 24];105(3):364–81. Available from: 10.1111/bjop.12043 25040006

[25] Perrett DI, Lee KJ, Penton-Voak I, Rowland D, Yoshikawa S, Burt DM, et al Effects of sexual dimorphism on facial attractiveness. Nature. 1998;394(6696):884–7. 10.1038/29772 9732869

[26] Rennels JL, Bronstad PM, Langlois JH. Are attractive men’s faces masculine or feminine? The importance of type of facial stimuli. J Exp Psychol Hum Percept Perform [Internet]. 2008 [cited 2018 Jul 24];34(4):884–93. Available from: http://doi.apa.org/getdoi.cfm?doi=10.1037/0096-1523.34.4.884 18665733 10.1037/0096-1523.34.4.884

[27] Zaidi A, White J, Mattern B, Liebowitz C, Puts D, Claes P et al Facial masculinity does not appear to be a condition-dependent male ornament and does not reflect MHC heterozygosity in humans. Proceedings of the National Academy of Sciences. 2019;116(5):1633–1638. 10.1073/pnas.1808659116 PMC6358690 30647112

[28] Keating CF. Gender and the Physiognomy of Dominance and Attractiveness. Soc Psychol Q [Internet]. 1985;48(1):61 Available from: http://www.jstor.org/stable/3033782?origin=crossref

[29] Swaddle JP, Reierson GW. Testosterone increases perceived dominance but not attractiveness in human males. Proc R Soc B Biol Sci. 2002;269(1507):2285–9. 10.1098/rspb.2002.2165 PMC1691166 12495494

[30] Langlois J, Roggman L. Attractive Faces Are Only Average. Psychological Science. 1990;1(2):115–121.

[31] Rhodes G, Tremewan T. Understanding face recognition: Caricauture effects, inversion, and the homogeneity problem. Vis cogn [Internet]. Taylor & Francis Group; 1994 4 24 [cited 2018 Jul 24];1(2–3):275–311. Available from: https://www.tandfonline.com/doi/full/10.1080/13506289408402303

[32] Rhodes G, Sumich A, Byatt G. Are Average Facial Configurations Attractive Only Because of Their Symmetry? Psychol Sci [Internet]. 1999 1 6 [cited 2018 Jul 24];10(1):52–8. Available from: http://journals.sagepub.com/doi/10.1111/1467-9280.00106

[33] Lie HC, Rhodes G, Simmons LW. GENETIC DIVERSITY REVEALED IN HUMAN FACES. Evolution (N Y) [Internet]. 2008 10 [cited 2018 Jul 24];62(10):2473–86. Available from: http://www.ncbi.nlm.nih.gov/pubmed/18691260 10.1111/j.1558-5646.2008.00478.x 18691260

[34] Roberts SC, Little AC, Gosling LM, Jones BC, Perrett DI, Carter V, et al MHC-assortative facial preferences in humans. Biol Lett. 2005;1(4):400–3. 10.1098/rsbl.2005.0343 17148217 PMC1626373

[35] Rhodes G, Yoshikawa S, Clark A, Kieran L, McKay R, Akamatsu S. Attractiveness of facial averageness and symmetry in non-western cultures: In search of biologically based standards of beauty. Perception. 2001;30(5):611–25. 10.1068/p3123 11430245

[36] Scheib JE, Gangestad SW, Thornhill R. Facial attractiveness, symmetry and cues of good genes. Proceedings Biol Sci [Internet]. The Royal Society; 1999 9 22 [cited 2018 Jul 24];266(1431):1913–7. Available from: http://www.ncbi.nlm.nih.gov/pubmed/10535106 10.1098/rspb.1999.0866 PMC1690211 10535106

[37] Thornhill R, Gangestad SW. Human facial beauty. Hum Nat [Internet]. 1993 9 [cited 2018 Jul 24];4(3):237–69. Available from: http://www.ncbi.nlm.nih.gov/pubmed/24214366 10.1007/BF02692201 24214366

[38] Lee AJ, Mitchem DG, Wright MJ, Martin NG, Keller MC, Zietsch BP. Facial averageness and genetic quality: Testing heritability, genetic correlation with attractiveness, and the paternal age effect. Evol Hum Behav [Internet]. Elsevier Inc.; 2016;37(1):61–6. Available from: 10.1016/j.evolhumbehav.2015.08.003 26858521 PMC4743547

[39] DeBruine LM, Jones BC, Unger L, Little AC, Feinberg DR. Dissociating Averageness and Attractiveness: Attractive Faces Are Not Always Average. J Exp Psychol Hum Percept Perform. 2007;33(6):1420–30. 10.1037/0096-1523.33.6.1420 18085954

[40] Said CP, Todorov A. A statistical model of facial attractiveness. Psychol Sci. 2011;22(9):1183–90. 10.1177/0956797611419169 21852448

[41] Alley TR, Cunningham MR. Article Commentary: Averaged Faces Are Attractive, but Very Attractive Faces Are Not Average. Psychol Sci [Internet]. SAGE PublicationsSage CA: Los Angeles, CA; 1991 3 25 [cited 2018 Jul 23];2(2):123–5. Available from: http://journals.sagepub.com/doi/10.1111/j.1467-9280.1991.tb00113.x

[42] Baudouin JY, Tiberghien G. Symmetry, averageness, and feature size in the facial attractiveness of women. Acta Psychol (Amst). 2004;117(3):295–312. 15500809 10.1016/j.actpsy.2004.07.002

[43] Jones D. Sexual Selection, Physical Attractiveness, and Facial Neoteny. 1995;36(December).

[44] Perrett DI, May KA, Yoshikawa S. Facial shape and judgements of female attractiveness. Nature [Internet]. Nature Publishing Group; 1994 3 17 [cited 2018 Jul 23];368(6468):239–42. Available from: http://www.nature.com/doifinder/10.1038/368239a0 8145822 10.1038/368239a0

[45] Wehr P, MacDonald K, Lindner R, Yeung G. Stabilizing and directional selection on facial paedomorphosis. Hum Nat [Internet]. Springer-Verlag; 2001 12 [cited 2018 Jul 23];12(4):383–402. Available from: http://link.springer.com/10.1007/s12110-001-1004-z 26192413 10.1007/s12110-001-1004-z

[46] Özener B, Fink B. Facial symmetry in young girls and boys from a slum and a control area of Ankara, Turkey. Evol Hum Behav [Internet]. Elsevier; 2010 11 1 [cited 2018 Jul 24];31(6):436–41. Available from: http://linkinghub.elsevier.com/retrieve/pii/S109051381000070X

[47] Thornhill R, Gangestad SW. Human Fluctuating Asymmetry and Sexual Behavior. Psychol Sci [Internet]. SAGE PublicationsSage CA: Los Angeles, CA; 1994 9 6 [cited 2018 Jul 24];5(5):297–302. Available from: http://journals.sagepub.com/doi/10.1111/j.1467-9280.1994.tb00629.x

[48] Banks GC, Batchelor JH, Mcdaniel MA. Intelligence Smarter people are (a bit) more symmetrical: A meta-analysis of the relationship between intelligence and fl uctuating asymmetry. Intelligence [Internet]. Elsevier Inc.; 2010;38(4):393–401. Available from: 10.1016/j.intell.2010.04.003

[49] Jones BC, Little AC, Tiddeman BP, Burt DM, Perrett DI. Facial symmetry and judgements of apparent health Support for a ‘“good genes”’ explanation of the attractiveness–symmetry relationship. 2001;22:417–29.

[50] Muñoz-Reyes JA, Iglesias-Julios M, Pita M, Turiegano E. Facial Features: What Women Perceive as Attractive and What Men Consider Attractive. PLoS One [Internet]. 2015 1 [cited 2017 Feb 11];10(7):e0132979 Available from: http://www.pubmedcentral.nih.gov/articlerender.fcgi?artid=4498779&tool=pmcentrez&rendertype=abstract 10.1371/journal.pone.0132979 26161954 PMC4498779

[51] Farrera A, Villanueva M, Quinto-Sánchez M, González-José R. The relationship between facial shape asymmetry and attractiveness in Mexican students. Am J Hum Biol. 2015;27(3):387–96. 10.1002/ajhb.22657 25400276

[52] Penton-Voak IS, Jones BC, Little AC, Baker S, Tiddeman B, Burt DM, et al Symmetry, sexual dimorphism in facial proportions and male facial attractiveness. Proc R Soc B Biol Sci. 2001;268(1476):1617–23. 10.1098/rspb.2001.1703 PMC1088785 11487409

[53] Soler C, Kekäläinen J, Núñez M, Sancho M, Núñez J, Yaber I, et al Male Facial Anthropometry and Attractiveness. Perception [Internet]. 2012 10 [cited 2017 Feb 11];41(10):1234–45. Available from: http://journals.sagepub.com/doi/10.1068/p7214 23469703 10.1068/p7214

[54] Van Dongen S. Associations between asymmetry and human attractiveness: Possible direct effects of asymmetry and signatures of publication bias. Ann Hum Biol [Internet]. 2011 5 [cited 2017 Feb 11];38(3):317–23. Available from: http://www.ncbi.nlm.nih.gov/pubmed/21271817 10.3109/03014460.2010.544676 21271817

[55] Rhodes G, & Simmons LW. Symmetry, attractiveness and sexual selection In: Dunbar RIM, Barrett L, eds. The Oxford handbook of evolutionary psychology. Oxford: University Press; 2007 p. 333–364.

[56] Fink B, Matts PJ, D’Emiliano D, Bunse L, Weege B, Röder S. Colour homogeneity and visual perception of age, health and attractiveness of male facial skin. J Eur Acad Dermatology Venereol [Internet]. Wiley/Blackwell (10.1111); 2011 11 [cited 2018 Jul 24];26(12):no-no. Available from: http://doi.wiley.com/10.1111/j.1468-3083.2011.04316.x 10.1111/j.1468-3083.2011.04316.x 22044626

[57] Lefevre CE, Perrett DI. Fruit over sunbed: Carotenoid skin colouration is found more attractive than melanin colouration. Q J Exp Psychol [Internet]. 2015 2 [cited 2018 Jul 24];68(2):284–93. Available from: http://www.ncbi.nlm.nih.gov/pubmed/25014019 10.1080/17470218.2014.944194 25014019

[58] Kleisner K, Kočnar T, Tureček P, Stella D, Akoko RM, Třebický V, et al African and European perception of African female attractiveness. Evol Hum Behav. 2017;38(6):744–55.

[59] Bovet J, Barthes J, Durand V, Raymond M, Alvergne A. Men’s Preference for Women’s Facial Features: Testing Homogamy and the Paternity Uncertainty Hypothesis. PLoS One. 2012;7(11). 10.1371/journal.pone.0049791 PMC3504097 23185437

[60] Edwards M, Cha D, Krithika S, Johnson M, Cook G, Parra EJ. Iris pigmentation as a quantitative trait: Variation in populations of European, East Asian and South Asian ancestry and association with candidate gene polymorphisms. Pigment Cell Melanoma Res. 2016;29(2):141–62. 10.1111/pcmr.12435 26547379

[61] Frost P. European hair and eye color. A case of frequency-dependent sexual selection? Evol Hum Behav. 2006;27(2):85–103.

[62] Frost P. The Puzzle of European Hair, Eye, and Skin Color. Adv Anthropol [Internet]. 2014;04(02):78–88. Available from: http://www.scirp.org/journal/doi.aspx?DOI = 10.4236/aa.2014.42011

[63] Jablonski NG, Chaplin G. The colours of humanity: The evolution of pigmentation in the human lineage. Philos Trans R Soc B Biol Sci. 2017;372(1724). 10.1098/rstb.2016.0349 PMC5444068 28533464

[64] Janif ZJ, Brooks RC, Dixson BJ. Negative frequency-dependent preferences and variation in male facial hair. Biol Lett. 2014;10(4). 10.1098/rsbl.2013.0958 PMC4013690 24740903

[65] Thelen TH. Minority type human mate preference. Biodemography Soc Biol [Internet]. 1983;30(2):162–80. Available from: http://www.tandfonline.com/doi/abs/10.1080/19485565.1983.9988531 10.1080/19485565.1983.9988531 6680247

[66] Fink B, Hufschmidt C, Hirn T, Will S, McKelvey G, Lankhof J. Age, health and attractiveness perception of virtual (rendered) human hair. Front Psychol. 2016;7(DEC):1–12. 28066276 10.3389/fpsyg.2016.01893 PMC5177627

[67] Janif ZJ, Brooks RC, Dixson BJ. Are Preferences for Women’s Hair Color Frequency-Dependent? Adapt Hum Behav Physiol. 2015;1(1):54–71.

[68] Lawson ED. Hair Color, Personality, and the Observer. Psychol Rep [Internet]. SAGE PublicationsSage CA: Los Angeles, CA; 1971 2 1 [cited 2018 Jul 24];28(1):311–22. Available from: http://journals.sagepub.com/doi/10.2466/pr0.1971.28.1.311 5549452 10.2466/pr0.1971.28.1.311

[69] Sorokowski P. Attractiveness of Blonde Women in Evolutionary Perspective: Studies with Two Polish Samples. Percept Mot Skills [Internet]. SAGE PublicationsSage CA: Los Angeles, CA; 2008 6 1 [cited 2018 Jul 24];106(3):737–44. Available from: http://journals.sagepub.com/doi/10.2466/pms.106.3.737-744 18712194 10.2466/pms.106.3.737-744

[70] Swami V, Barrett S. British men’s hair color preferences: An assessment of courtship solicitation and stimulus ratings. Scand J Psychol. 2011;52(6):595–600. 10.1111/j.1467-9450.2011.00911.x 21883260

[71] Bressan P, Damian V. Fathers’ eye colour sways daughters’ choice of both long- and short-term partners. Sci Rep [Internet]. Springer US; 2018;8(1):1–9. Available from: 10.1038/s41598-018-23784-7 29615697 PMC5883032

[72] Little AC, Penton-Voak IS, Burt DM, Perrett DI. Investigating an imprinting-like phenomenon in humans partners and opposite-sex parents have similar hair and eye colour. Evol Hum Behav. 2003;24(1):43–51.

[73] Wilson GD, Barrett PT. Parental characteristics and partner choice: Some evidence for oedipal imprinting. J Biosoc Sci. 1987;19(2):157–61. 10.1017/s0021932000016758 3584173

[74] Laeng B, Mathisen R, Johnsen JA. Why do blue-eyed men prefer women with the same eye color? Behav Ecol Sociobiol. 2007;61(3):371–84.

[75] Salter F. Carrier females and sender males: An evolutionary hypothesis linking female attractiveness, family resemblance, and paternity confidence. Ethol Sociobiol. 1996;17(4):211–20.

[76] Rantala MJ, Marcinkowska UM. The role of sexual imprinting and the Westermarck effect in mate choice in humans. Behav Ecol Sociobiol. 2011;65(5):859–73.

[77] Prokop P, Obertová Z, Fedor P. Paternity cues and mating opportunities: what makes fathers good?. acta ethologica. 2010;13(2):101–107.

[78] Kleisner K, Kočnar T, Rubešová A, Flegr J. Eye color predicts but does not directly influence perceived dominance in men. Pers Individ Dif. 2010;49(1):59–64.

[79] Kleisner K, Priplatova L, Frost P, Flegr J. Trustworthy-Looking Face Meets Brown Eyes. PLoS One. 2013;8(1):1–7. 10.1371/journal.pone.0053285 PMC3541379 23326406

[80] Hinsz VB, Stoesser CJ, Matz DC. The Intermingling of Social and Evolutionary Psychology Influences on Hair Color Preferences. Curr Psychol. 2013;32(2):136–49.

[81] Swami V, Rozmus-Wrzesinska M, Voracek M, Haubner T, Danel D, Pawłowski B, et al The influence of skin tone, body weight, and hair colour on perceptions of women’s attractiveness and health: A cross-cultural investigation. J Evol Psychol [Internet]. 2008;6(4):321–41. Available from: http://www.akademiai.com/doi/abs/10.1556/JEP.6.2008.4.4

[82] Moore FR, Coetzee V, Contreras-Garduño J, Debruine LM, Kleisner K, Krams I, et al Cross-cultural variation in women’s preferences for cues to sex- and stress-hormones in the male face. Biol Lett [Internet]. The Royal Society; 2013 6 23 [cited 2018 Jul 24];9(3):20130050 Available from: http://www.ncbi.nlm.nih.gov/pubmed/23536442 23536442 10.1098/rsbl.2013.0050 PMC3645036

[83] DeBruine LM, Jones BC, Crawford JR, Welling LLM, Little AC. The health of a nation predicts their mate preferences: Cross-cultural variation in women’s preferences for masculinized male faces. Proc R Soc B Biol Sci. 2010;277(1692):2405–10. 10.1098/rspb.2009.2184 PMC2894896 20236978

[84] DeBruine LM, Jones BC, Little AC, Crawford JR, Welling LLM. Further evidence for regional variation in women’s masculinity preferences. Proc R Soc B Biol Sci [Internet]. The Royal Society; 2011 3 22 [cited 2018 Jul 24];278(1707):813–4. Available from: http://rspb.royalsocietypublishing.org/cgi/doi/10.1098/rspb.2010.2200

[85] DeBruine LM, Little AC, Jones BC. Extending parasite-stress theory to variation in human mate preferences. Behav Brain Sci [Internet]. 2012 4 [cited 2018 Jul 24];35(02):86–7. Available from: http://www.ncbi.nlm.nih.gov/pubmed/22289354 22289354 10.1017/S0140525X11000987

[86] Marcinkowska UM, Kozlov MV, Cai H, Contreras-Garduño J, Barnaby J, Oana GA, et al Cross-cultural variation in men’s preference for sexual dimorphism in women’s faces Cross-cultural variation in men’s preference for sexual dimorphism in women’s faces. Biol Lett. 2014;10:2013–6. 10.1098/rsbl.2013.0850 PMC4013689 24789138

[87] Marcinkowska U, Rantala M, Lee A, Kozlov M, Aavik T, Cai H et al Women’s preferences for men’s facial masculinity are strongest under favorable ecological conditions. Scientific Reports. 2019;9(1). 10.1038/s41598-019-39350-8 PMC6399235 30833635

[88] Rhodes G, Chan J, Zebrowitz LA, Simmons LW. Does sexual dimorphism in human faces signal health? Proc R Soc B Biol Sci [Internet]. 2003 8 7 [cited 2018 Aug 6];270(Suppl_1):S93–5. Available from: http://www.ncbi.nlm.nih.gov/pubmed/12952647 10.1098/rsbl.2003.0023 PMC1698019 12952647

[89] Scott IML, Clark AP, Boothroyd LG, Penton-Voak IS. Do men’s faces really signal heritable immunocompetence? Behav Ecol. 2013;24(3):579–89. 10.1093/beheco/ars092 23555177 PMC3613940

[90] Dixson BJ, Little AC, Dixson HG, Brooks RC. Do prevailing environmental factors influence human preferences for facial morphology? Behav Ecol. 2017;28(5):1217–27.

[91] Little AC, Apicella CL, Marlowe FW. Preferences for symmetry in human faces in two cultures: Data from the UK and the Hadza, an isolated group of hunter-gatherers. Proc R Soc B Biol Sci. 2007;274(1629):3113–7. 10.1098/rspb.2007.0895 PMC2293939 17925281

[92] Apicella CL, Little AC, Marlowe FW. Facial averageness and attractiveness in an isolated population of hunter-gatherers. Perception. 2007;36(12):1813–20. 10.1068/p5601 18283931

[93] Little AC. Facial appearance and leader choice in different contexts: Evidence for task contingent selection based on implicit and learned face-behaviour/face-ability associations. Leadersh Q [Internet]. Elsevier Inc.; 2014;25(5):865–74. Available from: 10.1016/j.leaqua.2014.04.002

[94] Třebický V, Fialová J, Kleisner K, Havlíček J. Focal length affects depicted shape and perception of facial images. PLoS One. 2016;11(2):1–14. 10.1371/journal.pone.0149313 PMC4760932 26894832

[95] Danel DP, Dziedzic-Danel A, Kleisner K. Does age difference really matter? Facial markers of biological quality and age difference between husband and wife. HOMO- J Comp Hum Biol [Internet]. Elsevier GmbH.; 2016;67(4):337–47. Available from: 10.1016/j.jchb.2016.05.002 27238548

[96] Adams DC, Otárola‐Castillo E. geomorph: an r package for the collection and analysis of geometric morphometric shape data. Methods Ecol Evol [Internet]. Wiley/Blackwell (10.1111); 2013 [cited 2018 Jul 24];4(4):393–9. Available from: https://besjournals.onlinelibrary.wiley.com/doi/abs/10.1111/2041-210X.12035%4010.1111/%28ISSN%292041-210X.TOPMETHODS

[97] Mitteroecker P, Windhager S, Müller GB, Schaefer K. The Morphometrics of “Masculinity” in Human Faces. Raia P, editor. PLoS One [Internet]. 2015 2 11 [cited 2018 Jul 24];10(2):e0118374 Available from: http://www.ncbi.nlm.nih.gov/pubmed/25671667 10.1371/journal.pone.0118374 25671667 PMC4324773

[98] Klingenberg C, McIntyre G. Geometric Morphometrics of Developmental Instability: Analyzing Patterns of Fluctuating Asymmetry with Procrustes Methods. Evolution. 1998;52(5):1363 10.1111/j.1558-5646.1998.tb02018.x 28565401

[99] Mardia K, Bookstein F, Moreton I. ‘Statistical assessment of bilateral symmetry of shapes’. Biometrika. 2005;92(1):249–250.

[100] Klingenberg C, Barluenga M, Meyer A. SHAPE ANALYSIS OF SYMMETRIC STRUCTURES: QUANTIFYING VARIATION AMONG INDIVIDUALS AND ASYMMETRY. Evolution. 2002;56(10):1909–1920. 10.1111/j.0014-3820.2002.tb00117.x 12449478

[101] United Nations Development Programme. Human development report 2015. New York; 2015.

[102] Walsh S, Wollstein A, Liu F, Chakravarthy U, Rahu M, Seland JH, et al DNA-based eye colour prediction across Europe with the IrisPlex system. Forensic Sci Int Genet [Internet]. Elsevier Ireland Ltd; 2012;6(3):330–40. Available from: 10.1016/j.fsigen.2011.07.009 21813346

[103] Balaresque P, King TE. Human Phenotypic Diversity: An Evolutionary Perspective. Curr Top Dev Biol. 2016;119:349–90. 10.1016/bs.ctdb.2016.02.001 27282030

[104] Beals RL., Hoijer H. An introduction to anthropology. New York: Macmillan; 1965.

[105] Augood C, Fletcher A, Bentham G, Chakravarthy U, de Jong PTVM, Rahu M, et al Methods for a population-based study of the prevalence of and risk factors for age-related maculopathy and macular degeneration in elderly European populations: the EUREYE study. Ophthalmic Epidemiol [Internet]. 2004 4 [cited 2018 Jul 24];11(2):117–29. Available from: http://www.ncbi.nlm.nih.gov/pubmed/15255027 10.1076/opep.11.2.117.28160 15255027

[106] Kuznetsova A, Brockhoff P, Christensen R. lmerTest Package: Tests in Linear Mixed Effects Models. Journal of Statistical Software. 2017;82(13).

[107] Cunningham MR, Barbee AP, Pike CL. What do women want? Facialmetric assessment of multiple motives in the perception of male facial physical attractiveness. J Pers Soc Psychol [Internet]. 1990 [cited 2018 Jul 25];59(1):61–72. Available from: http://doi.apa.org/getdoi.cfm?doi=10.1037/0022-3514.59.1.61 2213490 10.1037//0022-3514.59.1.61

[108] Stirrat M, Perrett DI. Valid facial cues to cooperation and trust: Male facial width and trustworthiness. Psychol Sci. 2010;21(3):349–54. 10.1177/0956797610362647 20424067

[109] Stephen ID, Scott IML, Coetzee V, Pound N, Perrett DI, Penton-Voak IS. Cross-cultural effects of color, but not morphological masculinity, on perceived attractiveness of men’s faces. Evol Hum Behav. 2012;33(4):260–7.

[110] Puts DA. Beauty and the beast: mechanisms of sexual selection in humans. Evol Hum Behav [Internet]. 2010 5 [cited 2018 Jul 25];31(3):157–75. Available from: http://linkinghub.elsevier.com/retrieve/pii/S1090513810000279

[111] Lu HJ, Zhu XQ, Chang L. Good genes, good providers, and good fathers: Economic development involved in how women select a mate. Evol Behav Sci [Internet]. 2015 [cited 2018 Jul 25];9(4):215–28. Available from: http://doi.apa.org/getdoi.cfm?doi=10.1037/ebs0000048

[112] Lyons M, Marcinkowska U, Moisey V, Harrison N. The effects of resource availability and relationship status on women’s preference for facial masculinity in men: An eye-tracking study. Pers Individ Dif [Internet]. Elsevier Ltd; 2016;95:25–8. Available from: 10.1016/j.paid.2016.02.025

[113] Carrito M de L, Santos IMB dos, Lefevre CE, Whitehead RD, Silva CF da, Perrett DI. The role of sexually dimorphic skin colour and shape in attractiveness of male faces. Evol Hum Behav [Internet]. Elsevier Inc.; 2016;37(2):125–33. Available from: 10.1016/j.evolhumbehav.2015.09.006

[114] Rhodes G, Jeffery L, Watson TL, Clifford CWG, Nakayama K. Fitting the Mind to the World: Face Adaptation and Attractiveness Aftereffects. Psychol Sci. 2003;14(6):558–66. 10.1046/j.0956-7976.2003.psci_1465.x 14629686

[115] Abend P, Pflüger LS, Koppensteiner M, Coquerelle M, Grammer K. The sound of female shape: A redundant signal of vocal and facial attractiveness. Evol Hum Behav. 2015;36(3):174–81.

[116] Komori M, Kawamura S, Ishihara S. Averageness or symmetry: Which is more important for facial attractiveness? Acta Psychol (Amst) [Internet]. Elsevier B.V.; 2009;131(2):136–42. Available from: 10.1016/j.actpsy.2009.03.008 19394585

[117] Perrett DI, Burt DM, Penton-Voak IS, Lee KJ, Rowland DA, Edwards R. Symmetry and Human Facial Attractiveness. Evol Hum Behav [Internet]. Elsevier; 1999 9 1 [cited 2018 Jul 25];20(5):295–307. Available from: http://linkinghub.elsevier.com/retrieve/pii/S1090513899000148

[118] Pound N, Lawson DW, Toma AM, Richmond S, Zhurov AI, Penton-Voak IS. Facial fluctuating asymmetry is not associated with childhood ill-health in a large British cohort study. Proc R Soc B Biol Sci. 2014;281(1792). 10.1098/rspb.2014.1639 PMC4150332 25122232

[119] Van Dongen S. Associations among facial masculinity, physical strength, fluctuating asymmetry and attractiveness in young men and women. Ann Hum Biol. 2014;41(3):205–13. 10.3109/03014460.2013.847120 24555492

[120] Van Dongen S. Fluctuating asymmetry and masculinity/femininity in humans: A meta-analysis. Arch Sex Behav. 2012;41(6):1453–60. 10.1007/s10508-012-9917-7 22437551

[121] Van Dongen S, Gangestad SW. Human fluctuating asymmetry in relation to health and quality: a meta-analysis. Evol Hum Behav [Internet]. 2011 11 [cited 2018 Jul 25];32(6):380–98. Available from: http://linkinghub.elsevier.com/retrieve/pii/S1090513811000249

[122] Jones A, Jaeger B. Biological Bases of Beauty Revisited: The Effect of Symmetry, Averageness, and Sexual Dimorphism on Female Facial Attractiveness. Symmetry. 2019;11(2):279.

[123] Mogilski JK, Welling LLM. The Relative Importance of Sexual Dimorphism, Fluctuating Asymmetry, and Color Cues to Health during Evaluation of Potential Partners’ Facial Photographs: A Conjoint Analysis Study. Hum Nat. Human Nature; 2017;28(1):53–75. 10.1007/s12110-016-9277-4 27752965

[124] Graham J, Özener B. Fluctuating Asymmetry of Human Populations: A Review. Symmetry (Basel) [Internet]. 2016;8(12):154 Available from: http://www.mdpi.com/2073-8994/8/12/154

[125] Kavas S, Thornton A. Adjustment and hybridity in Turkish family change: Perspectives from developmental idealism. J Fam Hist. 2013;38(2):223–41.

[126] Sakallı-Uğurlu N. Quantitative Empirical Studies on Women’s Issues in Islamic Cultures: Introduction to Special Issue. Sex Roles. 2016;75(11–12):535–42.

[127] Yüksel-Kaptanoğlu İ, Ergöçmen BA. Early Marriage: Trends in Turkey, 1978–2008. J Fam Issues. 2014;35(12):1707–24.

[128] Karandashev V, Zarubko E, Artemeva V, Neto F, Surmanidze L, Feybesse C. Sensory Values in Romantic Attraction in Four Europeans Countries: Gender and Cross-Cultural Comparison. Cross-Cultural Res. 2016;50(5):478–504.

[129] Bánfai Z, Melegh B, Sümegi K, Hadzsiev K, Miseta A, Kásler M et al Revealing the Genetic Impact of the Ottoman Occupation on Ethnic Groups of East-Central Europe and on the Roma Population of the Area. Frontiers in Genetics. 2019;10. 10.3389/fgene.2019.00558 PMC6585392 31263480

[130] Iyigun M. Lessons from the Ottoman Harem on Culture, Religion, and Wars. Economic Development and Cultural Change. 2013;61(4):693–730.

[131] Deniz A., Özgür E.M. Rusya’dan Türkiye’ye ulus aşırı göç: Antalya’daki Rus göçmenler. Ege Coğrafya Dergisi 2010; 19(1): 13–30.

[132] Deniz A, Özgür E. Antalya'daki Rus Gelinler: Göçten Evliliğe, Evlilikten Göçe. İstanbul Üniversitesi Sosyoloji Dergisi. 2014; 3(27): 175–151.

[133] Davidjants B, Ross J. Conflicts in music in the South Caucasus: The case of Armenians and Azerbaijanis. Musicae Scientiae. 2016;21(4):430–441.

[134] Türkmenoğlu Berkan S, Manzakoğlu B. Evil Eye Belief in Turkish Culture: Myth of Evil Eye Bead. Turk Online J Des Art Commun. 2016;6(2):193–204.

[135] Perrett DI, Penton-Voak IS, Little AC, Tiddeman BP, Burt DM, Schmidt N, et al Facial attractiveness judgements reflect learning of parental age characteristics. Proc R Soc B Biol Sci. 2002;269(1494):873–80. 10.1098/rspb.2002.1971 PMC1690969 12028768

[136] Coetzee V, Greeff JM, Stephen ID, Perrett DI. Cross-cultural agreement in facial attractiveness preferences: The role of ethnicity and gender. PLoS One. 2014;9(7). 10.1371/journal.pone.0099629 PMC4079334 24988325

[137] Danel DP, Fedurek P, Coetzee V, Stephen ID, Nowak N, Stirrat M, et al A Cross-Cultural Comparison of Population-Specific Face Shape Preferences (Homo sapiens). Ethology. 2012;118(12):1173–81.

[138] Sorokowski P, Kościński K, Sorokowska A. Is beauty in the eye of the beholder but ugliness culturally universal? Facial preferences of polish and yali (papua) people. Evol Psychol. 2013;11(4):907–25.

[139] Rhodes G, Yoshikawa S, Palermo R, Simmonst LW, Peters M, Lee K, et al Perceived health contributes to the attractiveness of facial symmetry, averageness, and sexual dimorphism. Perception. 2007;36(8):1244–52. 10.1068/p5712 17972486

[140] GrayPB, FrederickDA. Body image and body type preferences in st. kitts, caribbean: A cross-cultural comparison with U.S. samples regarding attitudes towards muscularity, body fat, and breast size. Evol Psychol. 2012;10(3):631–55. 22995446

[141] Mo JJY, Cheung KWK, Gledhill LJ, Pollet T V., Boothroyd LG, Tovée MJ. Perceptions of Female Body Size and Shape in China, Hong Kong, and the United Kingdom. Cross-Cultural Res [Internet]. 2014;48(1):78–103. Available from: http://journals.sagepub.com/doi/10.1177/1069397113510272

[142] Tovée M, Swami V, Furnham A, Mangalparsad R. Changing perceptions of attractiveness as observers are exposed to a different culture☆. Evol Hum Behav [Internet]. 2006 11 [cited 2018 Jul 26];27(6):443–56. Available from: http://linkinghub.elsevier.com/retrieve/pii/S1090513806000584

[143] Swami V. Cultural Influences on Body Size Ideals. Eur Psychol [Internet]. Hogrefe Publishing; 2015 1 1 [cited 2018 Jul 26];20(1):44–51. Available from: http://econtent.hogrefe.com/doi/abs/10.1027/1016-9040/a000150

[144] Schneider TM, Hecht H, Stevanov J, Carbon CC. Cross-ethnic assessment of body weight and height on the basis of faces. Pers Individ Dif. Elsevier Ltd; 2013;55(4):356–60.

[145] Batres C, Kannan M, Perrett DI. Familiarity with Own Population’s Appearance Influences Facial Preferences. Hum Nat. Human Nature; 2017;28(3):344–54. 10.1007/s12110-017-9289-8 28516361 PMC5524856

[146] Thayer ZM, Dobson SD. Geographic Variation in Chin Shape Challenges the Universal Facial Attractiveness Hypothesis. PLoS One. 2013;8(4):1–5. 10.1371/journal.pone.0060681 PMC3616164 23560102

[147] Young SG, Hugenberg K, Bernstein MJ, Sacco DF. Perception and Motivation in Face Recognition: A Critical Review of Theories of the Cross-Race Effect. Personal Soc Psychol Rev. 2012;16(2):116–42. 10.1177/1088868311418987 21878608

[148] Brooks R, Scott IM, Maklakov AA, Kasumovic MM, Clark AP, Penton-Voak IS. National income inequality predicts women’s preferences for masculinized faces better than health does. Proceedings Biol Sci [Internet]. The Royal Society; 2011 3 22 [cited 2018 Jul 26];278(1707):810–2; discussion 813–4. Available from: http://www.ncbi.nlm.nih.gov/pubmed/21147809 10.1098/rspb.2010.0964 PMC3049041 21147809

[149] Batres C, Perrett DI. The influence of the digital divide on face preferences in El Salvador: People without internet access prefer more feminine men, more masculine women, and women with higher adiposity. PLoS One. 2014;9(7). 10.1371/journal.pone.0100966 PMC4089996 25006801

[150] Pollet T V., Tybur JM, Frankenhuis WE, Rickard IJ. What can cross-cultural correlations teach us about human nature? Hum Nat. 2014;25(3):410–29. 10.1007/s12110-014-9206-3 25092392

[151] Basu J, Ray R. Friends and Lovers: A Study of Human Mate Selection in India. Psychol -An Int J Psychol Orient [Internet]. Psychologia Society; 2001 [cited 2018 Jul 26];44(4):281–91. Available from: http://joi.jlc.jst.go.jp/JST.JSTAGE/psysoc/2001.281?from=CrossRef

[152] Muggleton NK, Fincher CL. Unrestricted sexuality promotes distinctive short- and long-term mate preferences in women. Pers Individ Dif. Elsevier Ltd; 2017;111:169–73.

[153] Bejanyan K, Marshall T, Ferenczi N. Romantic ideals, mate preferences, and anticipation of future difficulties in marital life: a comparative study of young adults in India and America. Frontiers in Psychology. 2014;5. 10.3389/fpsyg.2014.01355 PMC4251314 25520681

[154] Bugay A, Delevi R. “How can I say I love you to an American man and mean it?” Meaning of marriage among Turkish female students living in the U.S. Procedia—Social and Behavioral Sciences. 2010;5:1464–1470.

[155] Penton-Voak IS, Perrett DI, Castles DL, Kobayashi T, Burt DM, Murray LK, et al Menstrual cycle alters face preference. Nature [Internet]. Nature Publishing Group; 1999 6 24 [cited 2018 Jul 26];399(6738):741–2. Available from: http://www.nature.com/articles/21557 10.1038/21557 10391238

[156] Little AC, Jones BC, Penton-Voak IS, Burt DM, Perrett DI. Partnership status and the temporal context of relationships influence human female preferences for sexual dimorphism in male face shape. Proc R Soc B Biol Sci. 2002;269(1496):1095–100. 10.1098/rspb.2002.1984 PMC1691012 12061950

[157] Little AC, Cohen DL, Jones BC, Belsky J. Human preferences for facial masculinity change with relationship type and environmental harshness. Behav Ecol Sociobiol. 2007;61(6):967–73.

[158] Little AC, Mannion H. Viewing attractive or unattractive same-sex individuals changes self-rated attractiveness and face preferences in women. Anim Behav [Internet]. Academic Press; 2006 11 1 [cited 2018 Jul 26];72(5):981–7. Available from: https://www.sciencedirect.com/science/article/pii/S0003347206002697?via%3Dihub

[159] Boothroyd LG, Jones BC, Burt DM, DeBruine LM, Perrett DI. Facial correlates of sociosexuality. Evol Hum Behav [Internet]. 2008 5 [cited 2018 Jul 23];29(3):211–8. Available from: http://linkinghub.elsevier.com/retrieve/pii/S1090513808000032

[160] Frost P, Kleisner K, Flegr J. Health status by gender, hair color, and eye color: Red-haired women are the most divergent. PLOS ONE. 2017;12(12):e0190238 10.1371/journal.pone.0190238 29284020 PMC5746253

[161] Mitra S. ‘Miss World’ meets ‘dutiful daughter-in-law’: modernity, marriage, motherhood and the Bollywood female star. Celebrity Studies. 2018;10(2):228–246.

[162] Gelles, Rebecca. Fair and Lovely: Standards of Beauty, Globalization, and the Modern Indian Woman. Independent Study Project (ISP) Collection 2011; 1145.

